# Astrocyte Ca^2+^ in the dorsal striatum suppresses neuronal activity to oppose cue-induced reinstatement of cocaine seeking

**DOI:** 10.3389/fncel.2024.1347491

**Published:** 2024-08-29

**Authors:** Navid S. Tavakoli, Samantha G. Malone, Tanner L. Anderson, Ryson E. Neeley, Artin Asadipooya, Michael T. Bardo, Pavel I. Ortinski

**Affiliations:** ^1^Department of Neuroscience, University of Kentucky, Lexington, KY, United States; ^2^Department of Psychology, University of Kentucky, Lexington, KY, United States

**Keywords:** cocaine, reinstatement, astrocytes, dorsal striatum, calcium imaging, self-administration, medium spiny neuron

## Abstract

Recent literature supports a prominent role for astrocytes in regulation of drug-seeking behaviors. The dorsal striatum, specifically, is known to play a role in reward processing with neuronal activity that can be influenced by astrocyte Ca^2+^. However, the manner in which Ca^2+^ in dorsal striatum astrocytes impacts neuronal signaling after exposure to self-administered cocaine remains unclear. We addressed this question following over-expression of the Ca^2+^ extrusion pump, hPMCA2w/b, in dorsal striatum astrocytes and the Ca^2+^ indicator, GCaMP6f, in dorsal striatum neurons of rats that were trained to self-administer cocaine. Following extinction of cocaine-seeking behavior, the rats over-expressing hMPCA2w/b showed a significant increase in cue-induced reinstatement of cocaine seeking. Suppression of astrocyte Ca^2+^ increased the amplitude of neuronal Ca^2+^ transients in brain slices, but only after cocaine self-administration. This was accompanied by decreased duration of neuronal Ca^2+^ events in the cocaine group and no changes in Ca^2+^ event frequency. Acute administration of cocaine to brain slices decreased amplitude of neuronal Ca^2+^ in both the control and cocaine self-administration groups regardless of hPMCA2w/b expression. These results indicated that astrocyte Ca^2+^ control over neuronal Ca^2+^ transients was enhanced by cocaine self-administration experience, although sensitivity to acutely applied cocaine remained comparable across all groups. To explore this further, we found that neither the hMPCA2w/b expression nor the cocaine self-administration experience altered regulation of neuronal Ca^2+^ events by NPS-2143, a Ca^2+^ sensing receptor (CaSR) antagonist, suggesting that plasticity of neuronal signaling after hPMCA2w/b over-expression was unlikely to result from elevated extracellular Ca^2+^. We conclude that astrocyte Ca^2+^ in the dorsal striatum impacts neurons via cell-intrinsic mechanisms (e.g., gliotransmission, metabolic coupling, etc.) and impacts long-term neuronal plasticity after cocaine self-administration differently from neuronal response to acute cocaine. Overall, astrocyte Ca^2+^ influences neuronal output in the dorsal striatum to promote resistance to cue-induced reinstatement of cocaine seeking.

## Introduction

Cocaine use disorder is a significant public health issue characterized by compulsive drug-seeking and high rates of relapse (Kalivas and Volkow, 2005; Thomas et al., 2008). Relapse, the recurrence of drug-seeking behavior after a period of abstinence, poses a major challenge in the treatment of drug use (Sinha, 2011). Reinstatement of drug seeking is a widely used animal model that has been instrumental in the study of the neurobiological mechanisms underlying relapse (Kalivas and Volkow, 2005; Farrell et al., 2018) Recent reviews have highlighted evidence that astrocytes are actively involved in drug-seeking behaviors (Ortinski et al., 2022; Wang et al., 2022) and that astrocyte Ca^2+^ may exert significant influence on drug-use related cellular plasticity and behavioral alterations (Halassa and Haydon, 2010; Guerra-Gomes et al., 2017; Wang et al., 2022). Despite this evidence, the specific impact of astrocyte Ca^2+^ on neuronal plasticity that may underlie reinstatement of cocaine-seeking has not been previously evaluated.

In several studies, chemogenetic activation of G_i/o_- and G_q_-coupled GPCRs via viral vectors expressing designer receptors exclusively activated by designed drugs (DREADD) has been shown to regulate drug self-administration, habit-formation, and locomotion (Bull et al., 2014; Scofield et al., 2015; Nagai et al., 2019; Kang et al., 2020; Erickson et al., 2021). Variable effects of chemogenetic manipulations of astrocytes have been reported. For example, G_q_-DREADD stimulation of astrocytes in the prefrontal cortex (PFC) increased ethanol consumption in mice (Erickson et al., 2021). Meanwhile, G_q_-DREADD stimulation of astrocytes in the nucleus accumbens (NAc) core was found to decrease cue-induced reinstatement of cocaine seeking after extinction (Scofield et al., 2015). These results suggest potential differences in astrocyte contribution to drug-seeking across different substances, but may also indicate that the substantial heterogeneity of astrocytes between brain regions (Holt, 2023) supports different behavioral outcomes.

The dorsal striatum has been heavily implicated in reward processing (Balleine et al., 2007; Lewis et al., 2021), including the transition between goal-directed and habitual behaviors (Balleine et al., 2007; Lipton et al., 2019). Notably, recent work demonstrated that G_q_-DREADD stimulation of astrocytes in the dorsal striatum, elevates astrocyte Ca^2+^, suppresses glutamatergic output onto the D1 receptor-expressing medium spiny neurons (MSNs), and facilitates a transition from habitual to goal-directed sucrose-seeking (Kang et al., 2020). Another study specifically found that suppression of astrocyte Ca^2+^ in the dorsal striatum by over-expression of the Ca^2+^ extruder pump, hPMCA2w/b, led to development of repetitive (“compulsive”) behavioral stereotypies associated with reduced MSN action potential firing and spontaneous Ca^2+^ activity (Yu et al., 2018). Others have specifically linked astrocyte activity in the dorsal striatum to regulation of neuronal plasticity (Martín et al., 2015; Cavaccini et al., 2020). Therefore, dorsal striatal astrocyte Ca^2+^ may regulate MSN output and produce behavioral signatures relevant to substance use. However, it is not known whether drug experience affects the relationship between astrocyte Ca^2+^ and neuronal signaling in the dorsal striatum. In this study, we utilize hPMCA2w/b over-expression to evaluate whether cocaine self-administration alters the impact of astrocyte Ca^2+^ on MSN activity and explore the relevance of Ca^2+^ in the dorsal striatum astrocytes to cocaine self-administration behavior.

## Materials and methods

### Subjects

Male and female Sprague-Dawley rats (*N* = 30; 200–250 g) were single-housed under a 12/12-h light/dark cycle. All behavioral experiments were conducted during the light phase. Animals were subject to a 3-day acclimation period after arrival to the animal facility during which they were not handled and were provided with food and water, *ad libitum*. Following acclimation, the animals were handled daily until food restriction began. Animals began food restriction (~15–20 g/day for males, ~10–15 g/day for females) one day prior to their first cocaine self-administration session. Animals were weighed daily and body weight did not change by more than 20% through the course of experimentation in any of the animals. All laboratory procedures were reviewed and approved by the Institutional Animal Care and Use Communities at the University of Kentucky.

### Stereotaxic injections

Following isoflurane anesthesia (2–5% isoflurane in O_2_) subjects were secured in a stereotaxic apparatus (Kopf Instruments, Tujunga, CA, USA). All animals were injected bilaterally (1 μl/side) with the genetically encoded calcium indicator, GCaMP6f, targeting neurons (AAV9.Syn.GCaMP6f.WPRE.SV40, Addgene: 100837). This viral construct was originally described in Chen et al. (2013) and has since been extensively validated in numerous laboratories, including ours (O'Donovan et al., 2021). Half of the animals were injected bilaterally (2 μl/side) with the astrocyte-targeting plasma membrane Ca^2+^ ATPase pump (AAV5.gfaABC1D-mCherry-hPMCA2w/b, Addgene: 111568), originally described in Yu et al. (2018). The other half were injected with the astrocyte-targeting control virus (AAV5.gfaABC1D-tdTomato, Addgene: 44332; 2 μl/side), described in Shigetomi et al. (2013). Four animals received bilateral injections (2 μl/side) of astrocyte-specific GCaMP6f (AAV5.gfaABC1D-lck-GCaMP6f, Addgene: 52924) together with AAV-hPMCA2w/b (*N* = 2) or AAV-tdTomato (N=2) to confirm hPMCA2w/b-mediated suppression of astrocyte Ca^2+^. All viral injections were performed using a Neuros Syringe (Hamilton, Reno, NV, USA) at a rate of 0.2 μl/min, targeting the dorsal striatum at the following coordinates (relative to Bregma): A/P +0.5 mm; M/L ±3.0 mm; DV −4.5 mm. Animals were administered meloxicam (2 mg/kg, s.c.) for analgesia during surgery and once daily in the 48 h following the procedure and then given 10–14 days for recovery and to allow time for virus expression. Injection coordinates for all viruses were confirmed as termination of injection needle track in slice preparations.

### Jugular catheterization

Following recovery from virus injections, an indwelling silastic catheter (SAI Infusion Technologies, Lake Villa, IL., USA) was implanted into the right jugular vein under isoflurane anesthesia (2–5% isoflurane in O_2_). The catheter port was subcutaneously routed out and sutured in place between the shoulder blades. Catheters were flushed daily with 0.3 ml of antibiotic Timentin (0.93 mg/ml, Fisher, Hampton, NH, USA) dissolved in heparinized 0.9% saline and sealed with plastic obturators when not in use to prevent infection and maintain patency. Animals continued to be monitored daily for post-operative care and administered meloxicam (2 mg/kg, s.c.) during surgery and once daily in the 48 hours following the procedure. Animals were allowed 3–5 days of recovery before beginning self-administration.

### Immunohistochemistry

Three rats were transcardially perfused with 4% paraformaldehyde to evaluate extent and localization of AAV-tdTomato or AAV-hPMCA2w/b viruses in combination with astrocyte- or neuron-specific GCaMP6f using confocal microscopy. The brains were extracted and post-fixed with 4% paraformaldehyde for 24 h before transfer to 30% sucrose in distilled H_2_O. The brains were sliced into 40 μm sections using a cryostat and stored in cryoprotectant at −20°C. Striatal slices were washed in phosphate-buffered saline and 0.1% Triton (PBS+), followed by pre-blocking with 1.5% donkey serum in PBS+ for 60 min at room temperature. Subsequently, the slices were incubated overnight at 4°C with a 1:300 dilution of rabbit anti-GFP (Invitrogen GFP Polyclonal Antibody Thermofisher, Waltham, MA., USA Cat No. A-11122) and, for neuron identification, with a 1:500 dilution of mouse anti-NeuN (Anti-NeuN Antibody Millipore Sigma, Burlington, Massachusetts, USA Cat No. MAB377) in 1.5% donkey serum in PBS+. After washing with PBS+, the slices were incubated with secondary antibodies, 1:250 Donkey Anti-Rabbit (Alexa 488, Jackson ImmunoResearch Laboratories, West Grove, PA., USA Cat No. 711-545-152) and 1:250 Donkey Anti-Mouse (Alexa 647, Jackson ImmunoResearch Laboratories, West Grove, PA., USA Cat No. 715-605-151) as necessary in 1.5% donkey serum in PBS+ for 2 h at room temperature. Following another series of PBS+ washes, tissue was mounted on slides with DAPI Fluoromount-G (SouthernBiotech, Birmingham, AL., USA. Cat No. 0100-20) and then stored at 4°C until imaging. Images were acquired with 2x and 40x magnification objectives (Olympus FV3000, Center Valley, PA, USA).

### Cocaine self-administration, extinction, and reinstatement

All operant chambers were enclosed within a sound-attenuating box and controlled by the Med-PC IV software (Med Associates, St Albans, VT., USA). Each operant chamber contained two retractable levers and cue lights above each lever. Active lever pressing (right or left lever, counterbalanced between animals) during daily 1-h sessions was reinforced by cocaine infusions (0.2 mg cocaine/0.05 mL saline per each 2.8 s infusion) delivered via the implanted jugular catheter. Lever presses by control animals triggered infusion of bacteriostatic saline (0.05 mL per infusion). With every infusion, both cue lights were turned on for a total of 20 s. The cue lights signaled the time-out (TO) phase during which lever presses were counted as TO presses, but no additional infusions were delivered. Animals began with training on the fixed-ratio 1 (FR1) schedule of reinforcement (1 lever press = 1 infusion) for 7–10 days after which they were switched to the fixed-ratio 3 (FR3) schedule (3 lever presses = 1 infusion) for an additional 7 days of self-administration training. Following the last day of self-administration, the animals underwent 10 days of extinction training during 1 h daily sessions when lever responses did not trigger the infusions or cue light illumination. Following extinction training, the animals had a single 1 h session of cue-induced reinstatement under the FR1 schedule, during which active lever presses resulted in illumination of cue lights, but no infusions were administered.

### Brain slice preparation and Ca^2+^ imaging

The rats were deeply anesthetized with isoflurane and euthanized by decapitation between 24 and 48 h from reinstatement testing. Coronal brain slices (300 μm-thick) were prepared with a vibratome (VT1200S; Leica Microsystems, Wetzlar, Germany) in a chilled, continuously oxygenated (95% O_2_/5% CO_2_) artificial cerebrospinal fluid (aCSF) cutting solution containing the following (in mM): 93 NMDG, 2.5 KCl, 1.25 NaH_2_PO_4_, 30 NaHCO_3_, 20 HEPES, 25 glucose, 5 Na-ascorbate, 2 thiourea, 3 Na-pyruvate, 10 MgSO_4_, and 0.5 CaCl_2_ (adjusted to pH = 7.4 with NaOH, 300–310 mOsm). Slices recovered in the aCSF cutting solution at 34–36°C for 30 min during which aCSF-dissolved NaCl (2M) was introduced every 5 min in increasing volumes for a final NaCl concentration of 130 mM as previously described (Ting et al., 2018). Slices were then transferred to the aCSF recording solution, containing the following (in mM): 130 NaCl, 3 KCl, 1.25 NaH_2_PO_4_, 26 NaHCO_3_, 10 glucose, 1 MgCl_2_, and 2 CaCl_2_, pH 7.2–7.4, when saturated with 95% O_2_/5% CO_2_. Slices were maintained in the recording aCSF at room temperature until transfer to the imaging chamber, continually perfused with oxygenated recording aCSF (1.5–2.0 ml/min) at 31–33°C.

Spontaneous fluorescence of neuronal GCaMP6f was captured during 5 min videos (binned at 512 × 512 pixels) using the ORCA-Flash 4.0 (V2) digital camera (Hamamatsu, Shizuoka, Japan) under wide-field illumination with an LED light source (X-Cite XLED1, Excelitas Technologies, Miamisburg, OH, USA). The videos were acquired through a 40x objective (0.65 μm/pixel) at 25 frames per second. Twenty-one out of twenty-six behaviorally tested animals were subject to Ca^2+^ imaging at baseline. Some of these 21 animals were additionally subject to Ca^2+^ imaging in the presence of bath applied cocaine HCl (10 μM) and others—in the presence of the Ca^2+^-sensing receptor (CaSR) antagonist, NPS-2143 (100 nM), after acquisition of baseline fluorescence in the same field of view. Offline analyses of the videos were performed using ImageJ (U.S. National Institutes of Health, Bethesda, MD, USA) and MATLAB (Mathworks, Natick, MA, USA). Somatic regions of cells displaying spontaneous activity (i.e., active cells) were manually outlined by trained observers as regions of interest (ROIs) and fluorescence events were detected using the wavelet ridgewalking method (Neugornet et al., 2021). Somatic Ca^2+^ event amplitudes, durations, and frequencies, were extracted by custom-written MATLAB scripts and averaged for each identified ROI.

### Statistical analyses

All statistical analyses were performed using GraphPad Prism version 8.0 (GraphPad Software, San Diego, CA, USA). Descriptive statistics, including means, standard deviations, and frequencies, were calculated to summarize sample characteristics. The normality assumption of data distributions was assessed using the Shapiro-Wilk test. Two-way ANOVAs were followed by Fisher's LSD *post-hoc* comparisons. Non-parametric statistics were employed for analyses of non-normally distributed data. For non-normally distributed data comparing two groups, Mann-Whitney U tests were performed. For non-normally distributed data comparing more than two groups, Kruskal-Wallis H tests were performed, followed by Dunn's multiple comparisons if significant differences were detected. Wilcoxon matched- pairs tests were employed for non-parametric comparisons of paired data. All datasets have been analyzed for outliers using the ROUT method within GraphPad Prism (Q=1%). In all cases, removing the outliers did not change statistical interpretations of results and closer inspection of the data did not support non-biological origin of outlier events. Therefore, all outliers have been preserved in the final datasets. The significance threshold for all analyses was set at p < 0.05.

## Results

### Suppression of astrocyte Ca^2+^ in the dorsal striatum promotes cocaine seeking

**Figure 1:**
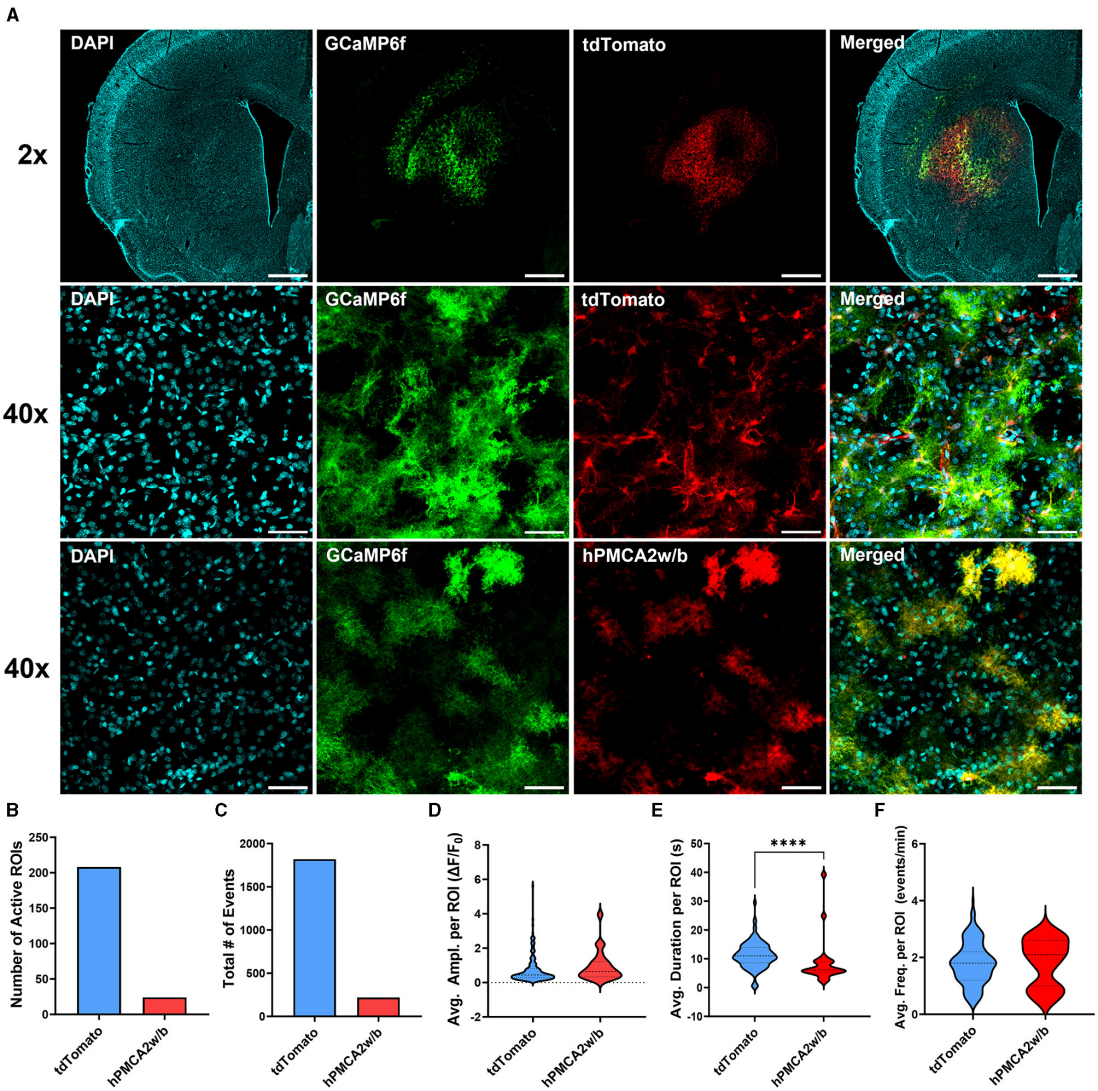
hPMCA2w/b over-expression suppresses astrocyte Ca^2+^ signaling in the dorsal striatum. **(A)** Individual and merged overlay confocal “z-stacks” (maximum intensity projections) of DAPI, astrocyte GCaMP6f, AAV-tdTomato, and AAV-hPMCA2w/b. *Top row*: 2x magnification images. Scale bar = 1 mm. *Middle/bottom rows*: 40x magnification images. Scale bar = 50 μm. **(B)** Total number of astrocytes displaying spontaneous Ca^2+^ events quantified as regions of interest (active ROIs) after transduction of the hPMCA2w/b or the control, tdTomato, virus. Note that the number of active ROIs likely underestimates the number of GCaMP6f-expressing astrocytes in both hPMCA2w/b and tdTomato groups since cells with static fluorescence could be detected in many of the imaged fields of view. **(C)** Total number of Ca^2+^ events observed across all cells in each group. **(D–F)** Amplitudes, durations, and frequencies of spontaneous Ca^2+^ events per ROI are displayed as ‘violin' plots. Plot width is proportional to frequency distributions of corresponding values. Dotted lines indicate the median and quartiles. **(B–F)** *N* = 2 tdTomato animals, 2 hPMCA2w/b animals, 5–9 slices in each group, *n* = 24–208 active cells in each group. ^****^p < 0.0001.

We began by examining astrocyte Ca^2+^ signaling in animals injected with AAV-hPMCA2w/b or the control AAV-tdTomato virus in combination with astrocyte-specific GCaMP6f (Figure 1A). Our results confirmed that hPMCA2w/b effectively suppressed spontaneous somatic Ca^2+^ activity in dorsal striatum astrocytes. In animals injected with the AAV-tdTomato, astrocyte Ca^2+^ transients were observed in 75% of imaged slices, whereas Ca^2+^ transients were observed in only 35% of all AAV-hPMCA2w/b slices. Expression of hPMCA2w/b reduced the number of active astrocytes and the total number of Ca^2+^ events across all cells (Figures 1B, C; Supplementary material 1). Notably, the few Ca^2+^ events that we were able to capture in astrocytes after hPMCA2w/b injection were not different from events in control astrocytes after tdTomato injection in either Ca^2+^ event amplitude or frequency (Figures 1D, F). However, hPMCA2w/b injection significantly reduced the duration of astrocyte Ca^2+^ events relative to the tdTomato group (Mann-Whitney; U = 1,034, *p* < 0.0001) (Figure 1E).

**Figure 2:**
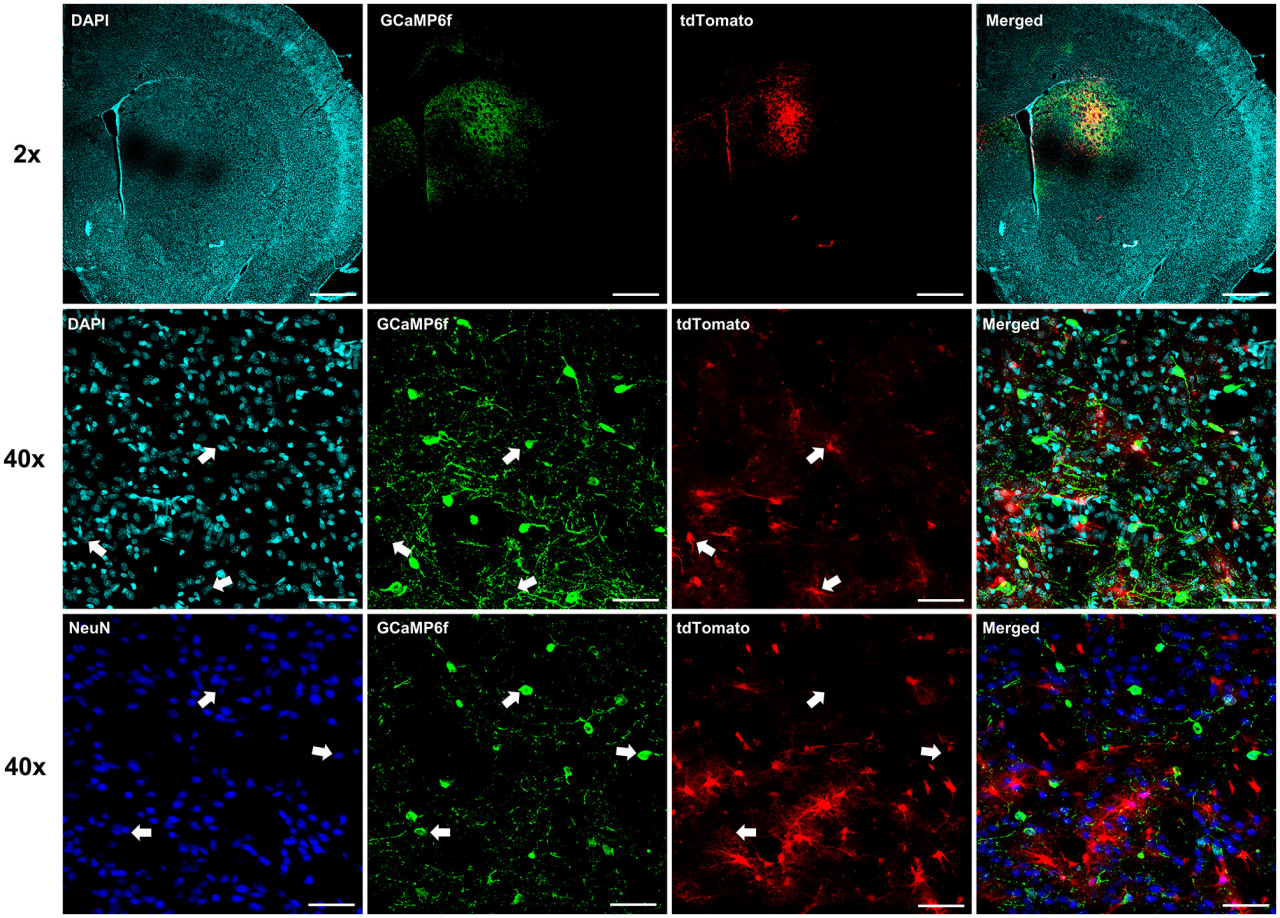
Expression of neuronal GCaMP6f. Individual and merged overlay confocal “z-stacks” (maximum intensity projections), illustrating segregation of neuronal GCaMP6f and astrocyte labels. *Top row*: 2x magnification images. Scale bar = 1 mm. *Middle row*: 40x magnification images with arrows indicating location of astrocyte-targeted tdTomato in different fluorescent channels. Note clear segregation of tdTomato and neuronal GCaMP6f labels. Scale bar = 50 μm. *Bottom row*: 40x magnification images with arrows indicating location of GCaMP6f-positive neuron somas. Again, note segregation with the astrocyte tdTomato, but overlap with the NeuN, labels. Scale bar = 50 μm.

**Figure 3:**
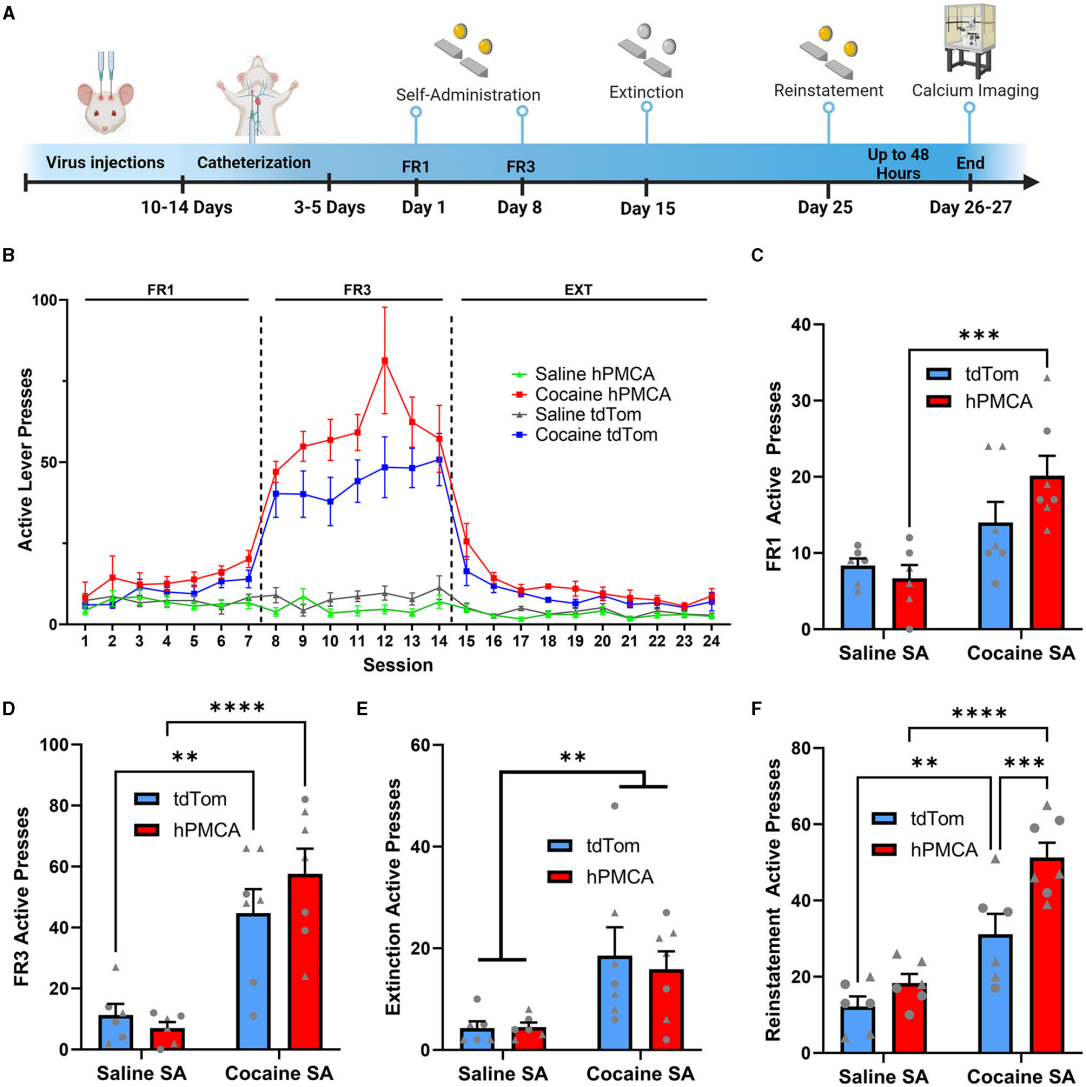
Suppression of astrocyte Ca^2+^ increases cocaine seeking during cue-induced reinstatement. **(A)** Experimental timeline of surgeries, behavioral procedures, and wide-field Ca^2+^ imaging in the slice. **(B)** Active presses across all days of self-administration and extinction training. **(C)** Histogram of active lever presses during the last day of FR1 training. **(D)** Histogram of active lever presses during the last day of FR3 training. **(E)** Histogram of total active lever presses during the last day of extinction training. Significance symbols indicate main effect of cocaine (two-way ANOVA). **(F)** Histogram of total active lever presses during cue-induced reinstatement. *N* = 6–7 animals in each group. Triangles—males, circles—females. **p < 0.001; ***p < 0.001; ****p < 0.0001. All graphs indicate mean ± SEM.

To investigate whether astrocyte Ca^2+^ in the dorsal striatum regulates cocaine seeking, animals expressing neuronal GCaMP6f and hPMCA2w/b or tdTomato (Figure 2) were trained to self-administer cocaine on FR1 and FR3 schedules of reinforcement, followed by 10 days of extinction training (Figures 3A, B). As expected, overall effects of time (session number) and drug self-administration as well as time x drug interactions were present during FR1, FR3, and extinction training, but there was no overall effect of hPMCA2w/b treatment across sessions or across drug administration regimes [FR1 time: F_(2, 54)_ = 3.432, *p* = 0.0311; FR1 drug: F_(1, 22)_ = 6.715, *p* = 0.0167; FR1 time x drug: F_(6, 132)_ = 2.895, *p* = 0.0110; FR3 time: F_(2.7, 56)_ = 4.254, *p* = 0.0110; FR3 drug: F_(1, 22)_ = 79.63, *p* < 0.0001; FR3 time x drug: F_(6, 124)_ = 3.317; *p* = 0.0046; extinction time: F_(2, 44)_ = 11.58, *p* < 0.0001; extinction drug: F_(1, 22)_ = 33.03, *p* < 0.0001; extinction time x drug: F_(9, 187)_ = 7.207, *p* < 0.0001; 3-way RM ANOVAs].

The experimental groups were further evaluated by comparing active lever presses in the final day of FR1 or FR3 training. On the last day of FR1 training, a two-way ANOVA did not reveal a significant interaction between cocaine exposure and hPMCA2w/b expression [F_(1, 22)_ = 3.029; p = 0.0958] while showing a significant main effect of cocaine exposure [F_(1, 22)_ = 18.20; p = 0.0003], but not hPMCA2w/b expression [F_(1, 22)_ = 0.9952; p = 0.3293]. *Post-hoc* comparisons (Fisher's LSD) indicated a significantly higher number of presses in the cocaine hPMCA2w/b group relative to the saline hPMCA2w/b group (t_22_ = 4.247, *p* = 0.0003). While trending, the cocaine tdTomato group did not significantly differ from the saline tdTomato saline group (t_22_ = 1.786, p = 0.0879). Likewise, elevated number of active presses in cocaine hPMCA2w/b animals failed to reach statistical significance relative to the cocaine tdTomato group at the end of FR1 training (t_22_ = 2.015, p = 0.0563). No significant differences were detected between saline tdTomato and saline hPMCA2w/b controls (t_22_ = 0.5062, *p* = 0.6178) (Figure 3C).

On the final FR3 training session, there was also no significant interaction between cocaine exposure and hPMCA2w/b expression [F_(1, 22)_ = 1.741; p = 0.2006, two-way ANOVA] while once again demonstrating a significant main effect of cocaine exposure [F_(1, 22)_ = 41.52; *p* < 0.0001], but not hPMCA2w/b expression [F_(1, 22)_ = 0.4280; p = 0.5197] *Post-hoc* analyses (Fisher's LSD) revealed a significantly greater number of lever presses in the cocaine hPMCA2w/b group relative to the saline hPMCA2w/b group (t_22_ = 5.489, *p* < 0.0001) as well as significantly more presses in the cocaine tdTomato group relative to the saline tdTomato group (t_22_ = 3.623, *p* = 0.0015). Meanwhile, there were no significant differences between saline tdTomato and saline hPMCA2w/b controls (t_22_ = 0.4533, *p* = 0.6548) and no significant differences were observed between the cocaine hPMCA2w/b groups and the cocaine tdTomato group when looking at the last day of FR3 (t_22_ = 1.453, *p* = 0.1605) (Figure 3D). Cocaine exposure and hPMCA2w/b expression had no impact on inactive lever presses (Supplementary Figure 1A). There was no interaction between cocaine exposure and hPMCA2w/b and no main effect of hPMCA2w/b treatment or cocaine exposure on inactive presses during the last day of FR1 and FR3 training (Supplementary Figures 1B, C).

Additionally, during extinction training there was a significant main effect of cocaine, wherein total lever pressing (including TO presses) in the last day of extinction was elevated in cocaine- vs. saline self-administering animals [F_(1, 22)_ = 12.27; p <0.0020]. However, cocaine hPMCA2w/b animals pressed on the active lever at similar levels to their cocaine tdTomato counterparts and there was no main effect of hPMCA2w/b treatment and no interaction between hPMCA2w/b treatment and cocaine exposure during the extinction training (Figure 3E). There was also no interaction between cocaine exposure and hPMCA2w/b and no main effect of hPMCA2w/b treatment or cocaine exposure on inactive presses on the last day of extinction training (Supplementary Figure 1D). We considered the possibilities that time-out pressing may differ across experimental conditions or that it may influence interpretation of reinforced active lever presses. However, no significant differences in time-out responses were detected during FR1, FR3, and extinction sessions.

We next evaluated the ability of cocaine-associated light cues to produce reinstatement of cocaine seeking. Significant main effects of both cocaine self-administration [F_(1, 21)_ = 47.29; p <0.0001] and hPMCA2w/b expression were detected [F_(1, 21)_ = 12.11; p = 0.0022], but there was no interaction between cocaine exposure and hPMCA2w/b expression [F_(1, 21)_ = 3.411; p = 0.0789; two-way ANOVA]. Cocaine hPMCA2w/b animals showed significantly increased cue-induced reinstatement compared to cocaine tdTomato group (t_21_ = 3.838, p = 0.0010, Fisher's LSD), with no significant differences between saline hPMCA2w/b and saline tdTomato groups (t_21_ = 1.134, p = 0.2698, Fisher's LSD). As expected, cue-induced reinstatement was elevated in the cocaine tdTomato relative to the saline tdTomato group (t_21_ = 3.493, p = 0.0022, Fisher's LSD) and in cocaine hPMCA2w/b relative to the saline hPMCA2w/b (t_21_ = 6.286, p <0.0001, Fisher's LSD) group (Figure 3F). For inactive lever presses, there was no interaction between cocaine exposure and hPMCA2w/b expression and no main effect of hPMCA2w/b expression during the reinstatement testing. However, there was a significant main effect of cocaine (F_(1, 21)_ = 6.096; p = 0.0022) with elevated inactive lever pressing in cocaine- vs. saline self-administering animals (Supplementary Figure 1E). Importantly, we found no correlation between active lever presses during the FR3 and reinstatement sessions within animals from either the tdTomato or the hPMCA2w/b groups (Supplementary Figure 2), supporting a dissociation between acquisition and reinstatement training results. Altogether these data indicate increased acquisition and cue-induced reinstatement of cocaine seeking after suppression of Ca^2+^ in astrocytes of the dorsal striatum, suggesting that astrocyte Ca^2+^ signaling in this brain area opposes expression of cocaine-seeking behavior.

### Suppression of astrocyte Ca^2+^ in the dorsal striatum increases neuronal Ca^2+^ signaling after cocaine self-administration

**Figure 4:**
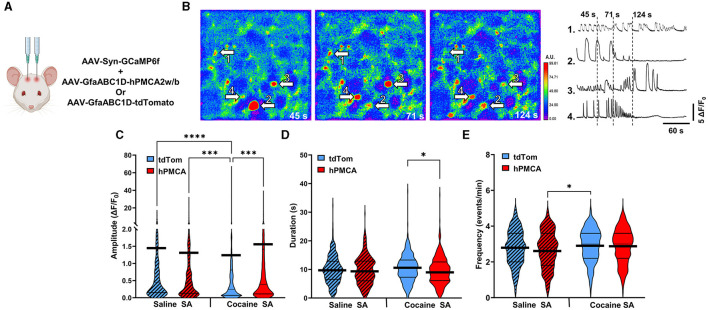
Suppression of astrocyte Ca^2+^ elevates amplitude of neuronal Ca^2+^ transients in the dorsal striatum following cocaine self-administration. **(A)** Schematic of virus injections. **(B)** *Left*: False-color heatmaps of neuronal Ca^2+^ fluorescence within the same field of view at time points indicated at bottom right of each panel. Areas of low activity are depicted in cooler colors and areas of high activity are depicted in warmer colors representing the arbitrary units (A.U.) of fluorescence marked in the scale bar to the right. The arbitrary units (A.U.) on the scale bar represent fractions of maximum fluorescence intensity across the entire field. *Right*: Representative ΔF/F_0_ traces, illustrating Ca^2+^ activity across the imaging session in select neurons indicated by arrows in **(A)**. **(C–E)** Violin plots of amplitudes, durations, and frequencies of spontaneous Ca^2+^ events. Thin black lines indicate medians and quartiles. Thick black lines are centered at the means. *N* = 4–6 animals in each group. 18–36 Slices in each group. *n* = 308–414 cells in each group. *p < 0.05; ***p < 0.0001; ****p < 0.0001.

We monitored spontaneous neuronal Ca^2+^ transients in brain slices prepared at 24-48 h after reinstatement testing by measuring fluorescence of neuron-targeting Ca^2+^ indicator, GCaMP6f, (Figures 4A, B, Supplementary material 1). There was a significant group effect on the amplitude of neuronal Ca^2+^ transients that has been typically interpreted to reflect action potential generation (Akerboom et al., 2012; Ali and Kwan, 2020; Zhang et al., 2023) (H = 34.53, df = 3, *p* < 0.0001; Kruskal-Wallis) (Figure 4C). Specifically, the amplitude of neuronal Ca^2+^ events was suppressed by cocaine experience in cocaine tdTomato, relative to the saline tdTomato, group (z = 5.456, *p* < 0.0001, Dunn's multiple comparisons test). In cocaine hPMCA2w/b animals, however, the amplitude of neuronal Ca^2+^ transients were significantly elevated relative to their cocaine tdTomato counterparts (z = 4.272, *p* = 0.0001, Dunn's test). There was no significant difference between saline tdTomato and saline hPMCA2w/b groups (z = 1.025, *p* > 0.9999, Dunn's test) and no difference between cocaine hPMCA2w/b and saline hPMCA2w/b groups (z = 0.1107, *p* > 0.9999, Dunn's test), indicating that hPMCA2w/b expression mitigates inhibitory effects of cocaine on amplitude of neuronal Ca^2+^ transients.

Our analyses also revealed significant group effects on duration (H = 8.527, df = 3, *p* = 0.0363) and frequency (H = 11.45, df = 3, *p* = 0.0095) of neuronal Ca^2+^ events. In the case of event duration data, this was driven by both the increased event duration in the cocaine tdTomato (relative to saline tdTomato) group and decreased event duration in the cocaine hPMCA2w/b (relative to saline hPMCA2w/b) group (z = 2.727, *p* = 0.0383, Dunn's test) (Figure 4D). In the case of event frequency data, the group effect was driven by higher event frequency in the cocaine tdTomato compared to the saline hPMCA2w/b group (z = 3.068, *p* = 0.0129, Dunn's test) (Figure 4E). The elevated frequency of Ca^2+^ events in cocaine hPMCA2w/b compared to the saline hPMCA2w/b group, failed to reach statistical significance (z = 2.549, *p* = 0.0648, Dunn's test).

Our finding that increased amplitude of neuronal Ca^2+^ events in hPMCA2w/b over-expressing animals was observed only after cocaine self-administration indicates that cocaine exposure influences the interaction between astrocyte and neuronal Ca^2+^ in the dorsal striatum. Moreover, reduced Ca^2+^ event amplitudes in cocaine tdTomato, relative to both saline tdTomato and cocaine hPMCA2w/b groups, suggest that astrocyte Ca^2+^ acts to suppress neuronal activity after cocaine self-administration and extinction.

### Acute administration of cocaine suppresses neuronal activity regardless of astrocyte Ca^2+^ or cocaine self-administration history

**Figure 5:**
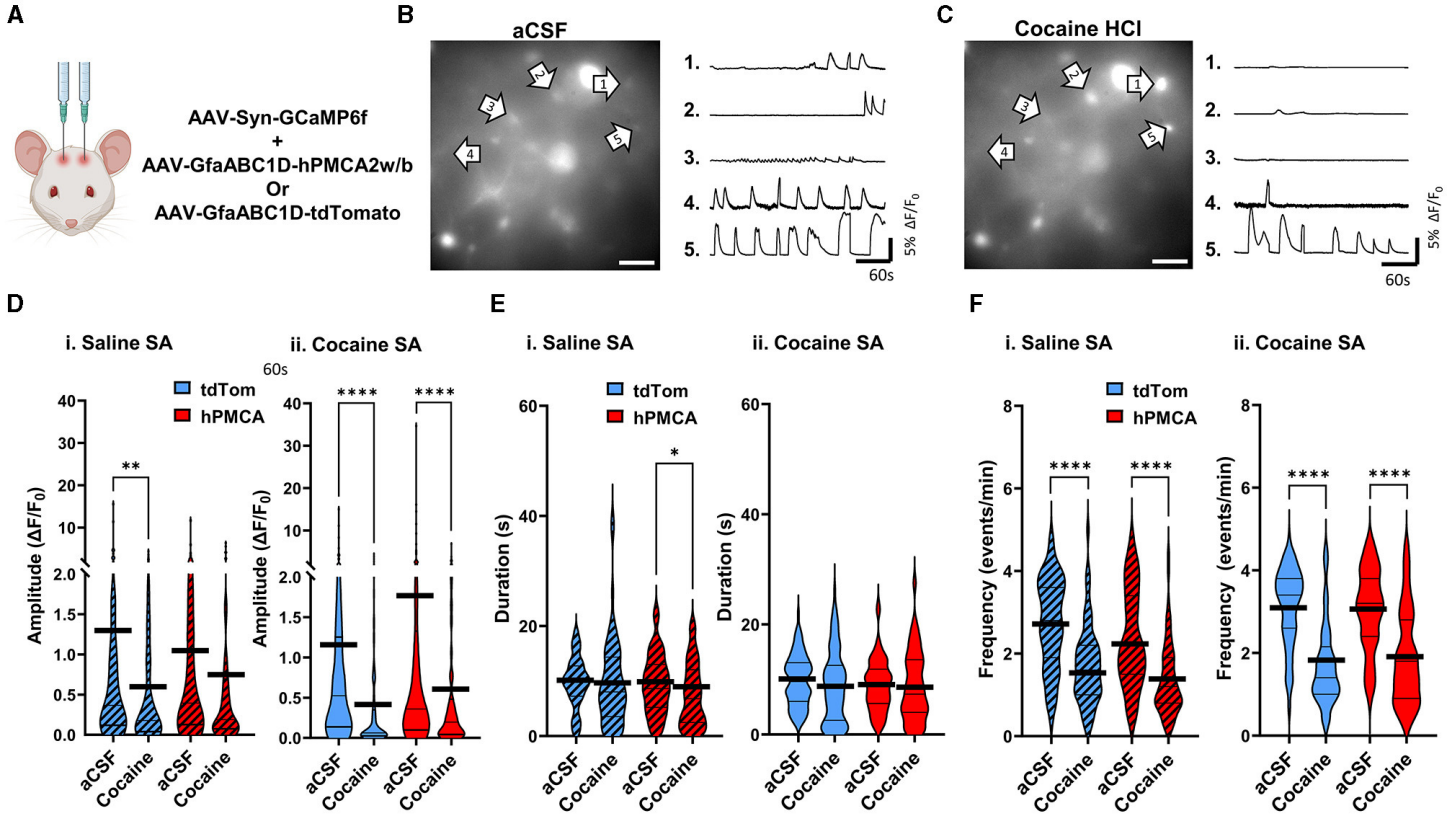
Acute application of cocaine broadly suppresses neuronal Ca^2+^ signaling. **(A)** Schematic of virus injections. **(B)** *Left*: A single video frame of raw neuronal Ca^2+^ fluorescence in the aCSF condition. Scale bar = 50 μM. *Right*: Representative ΔF/F_0_ traces, illustrating Ca^2+^ activity across the imaging session in select neurons indicated by arrows. **(C)** *Left*: Same field of view as in **(B)**, but after bath application of 10μM cocaine HCl. Scale bar = 50 μM. *Right*: Representative ΔF/F_0_ traces, illustrating Ca^2+^ activity in select neurons indicated by arrows. **(D–F)** Violin plots of neuronal Ca^2+^ event amplitudes, durations, and frequencies in saline self-administration (saline SA) and cocaine self-administration (cocaine SA) groups. Thin black lines indicate medians and quartiles. Thick black lines are centered at the means. *N* = 4–6 animals in each group. 9–21 FOVs in each group. *n* = 61–100 cells in each group. *p < 0.05; **p < 0.01; ****p < 0.0001.

To investigate whether cocaine effects on neuronal Ca^2+^ require self-administration of cocaine, acute cocaine was applied to slices from hPMCA2w/b and tdTomato groups (Figures 5A–C, Supplementary material 1 cf. Figure 1 for viral label expression). Baseline neuronal Ca^2+^ transients were recorded for 5 min after which cocaine (10 μM) was introduced to aCSF for 5 minutes, and neuronal activity was recorded in the same field of view for an additional 5 min. Acute cocaine suppressed the amplitude of neuronal Ca^2+^ events in the saline tdTomato (*p* = 0.0020, Wilcoxon matched-pairs test), cocaine tdTomato (*p* < 0.0001, Wilcoxon matched-pairs test), and cocaine hPMCA2w/b (*p* < 0.0001, Wilcoxon matched-pairs test) groups. Reduced values of neuronal Ca^2+^ amplitudes after acute cocaine in slices from saline hPMCA2w/b rats failed to clear the significance threshold (*p* = 0.0944, Wilcoxon). Acute cocaine effect size was reduced in hPMCA2w/b groups, relative to the tdTomato groups regardless of self-administration experience (saline tdTomato d = 0.36, saline hPMCA2w/b d = 0.20, cocaine tdTomato d = 0.47, cocaine hPMCA2w/b d = 0.35) (Figure 5D). Similar to findings with the Ca^2+^ event amplitude, acute cocaine application produced broadly similar effects on event duration, in this case, failing to alter this measure of neuronal Ca^2+^ in three out of four experimental groups. Again, the saline hPMCA2w/b group was a notable exception with decreased duration of neuronal Ca^2+^ transients after cocaine self-administration (*p* = 0.0334, Wilcoxon matched-pairs test). The effect sizes in the saline tdTomato (d = 0.03) and cocaine hPMCA2w/b (d = 0.05) groups were smaller than the effect sizes in saline hPMCA2w/b (d = 0.36) and cocaine tdTomato (d = 0.20) groups (Figure 5E). In the case of event frequency, acute cocaine prominently suppressed this measure of neuronal Ca^2+^ in each of the four experimental groups (*p* < 0.0001, Wilcoxon matched-pairs test). The effect sizes in the four groups were larger in the cocaine groups (hPMCA2w/b (d = 1.07); tdTomato (d = 1.47), than in the saline tdTomato (d = 0.98) and saline hPMCA2w/b (d = 0.94) groups (Figure 5F).

Overall, although larger effect sizes in amplitude and frequency measures were observed after cocaine self-administration training, this set of experiments failed to identify a unique effect of acute cocaine on neuronal Ca^2+^ events from cocaine tdTomato relative to cocaine hPMCA2w/b animals. Notably, these results suggest that potentiation of neuronal activity after suppression of Ca^2+^ in dorsal striatum astrocytes emerges as a consequence of cocaine self-administration rather than as reflection of hPMCA2w/b effect on neuronal response to acute cocaine.

### Increased neuronal activity after hPMCA2w/b over-expression and cocaine self-administration is not due to rise in extracellular Ca^2+^

**Figure 6:**
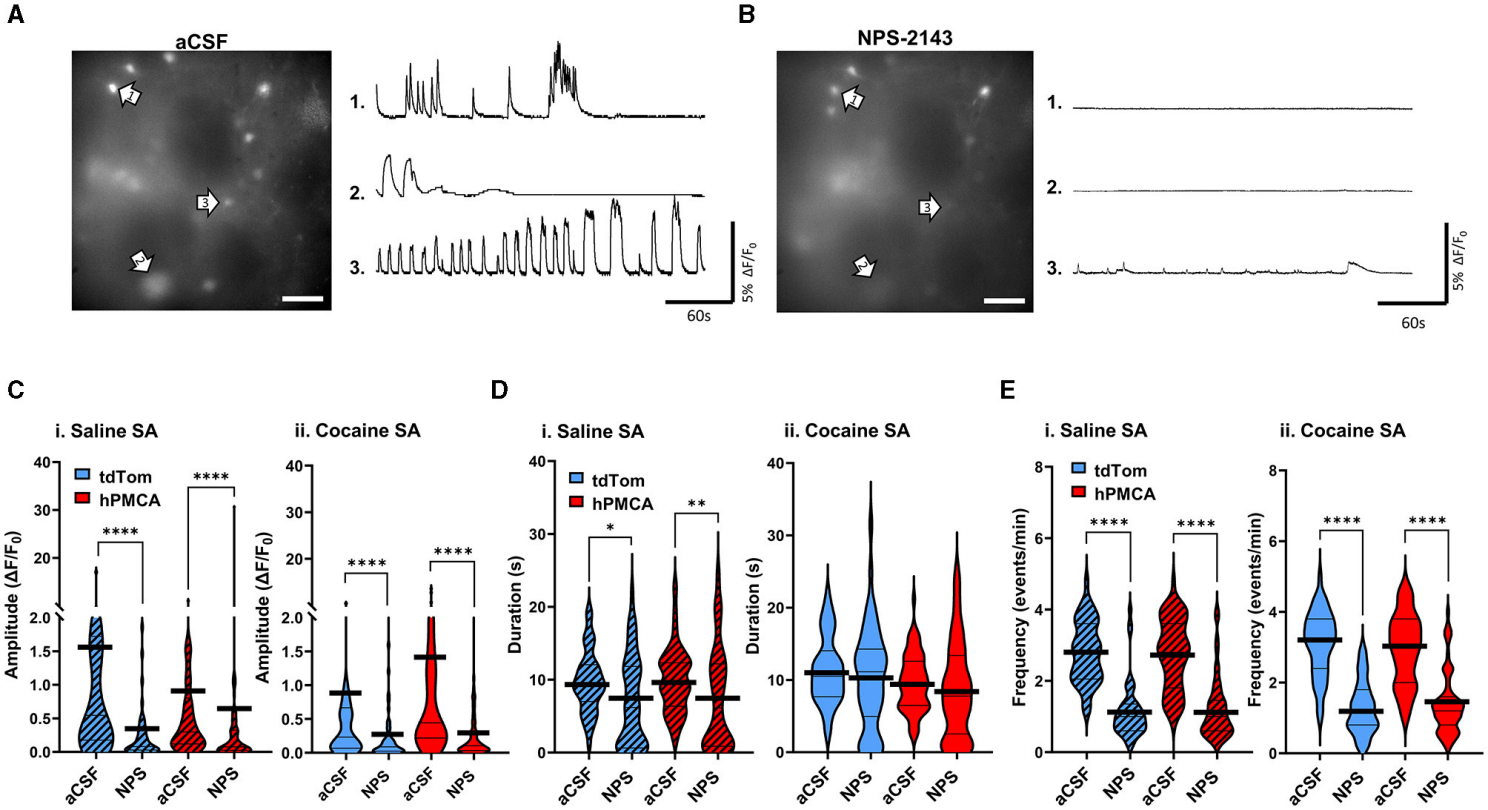
Inhibition of the CaSR has comparable effects on neuronal Ca^2+^ regardless of cocaine experience or hPMCA2w/b expression. **(A)** *Left*: A single video frame of raw neuronal Ca^2+^ fluorescence recorded in aCSF conditions. Scale bar = 50 μM. *Right*: Representative ΔF/F_0_ traces, illustrating Ca^2+^ activity across the imaging session in select neurons indicated by arrows. **(B)** *Left*: Same field of view as in **(A)**, but after bath application of 100nM NPS-2143. Scale bar = 50 μM. *Right*: Representative ΔF/F_0_ traces, illustrating Ca^2+^ activity across the imaging session in select neurons indicated by arrows in B). **(C–E)** Violin plots of Ca^2+^ event amplitudes, durations, and frequencies in saline self-administration (saline SA) and cocaine self-administration (cocaine SA) groups. N= 3-5 animals in each group. 8–12 Slices in each group. *n* = 48–67 cells in each group. Thin black lines indicate medians and quartiles. Thick black lines are centered at the means. *p < 0.05; **p < 0.01; ****p < 0.0001.

In the last set of experiments, we explored whether increased neuronal Ca^2+^ signals may have stemmed from increased extracellular Ca^2+^ levels due to continuous Ca^2+^ extrusion by the hPMCA2w/b. To do so, we leveraged the high sensitivity of the endogenous Ca^2+^-sensing receptor (CaSR) to extracellular Ca^2+^ levels (Gray and Golowasch, 2016). Binding of extracellular Ca^2+^ by CaSR leads to Ca^2+^ release from intracellular stores via activation of the G_q/11_ pathway (Lu et al., 2010; Thakker, 2012; Abid et al., 2021). We reasoned that if hPMCA2w/b elevated extracellular Ca^2+^ to impact neuronal activity, slices from hPMCA2/b animals would display elevated neuronal GCaMP6f sensitivity to CaSR inhibition. After a 5-min baseline recording of activity in aCSF (Figure 6A), the CaSR antagonist, NPS-2143 (100 nM), was introduced to the bath for 10 min, following which 5-min videos of spontaneous Ca^2+^ fluorescence were acquired in the same field of view (Figure 6B, Supplementary material 1). We observed a significant suppression of neuronal Ca^2+^ amplitude by NPS-2143 in all groups (*p* < 0.0001, Wilcoxon matched-pairs tests) regardless of cocaine self-administration experience or hPMCA2w/b expression. The effect sizes were d = 0.59, 0.07, 0.39, and 0.62 for saline tdTomato, saline hPMCA2w/b, cocaine tdTomato, and cocaine hPMCA2w/b groups, respectively (Figure 6C). Duration of neuronal Ca^2+^ events was significantly reduced by NPS-2143 in saline tdTomato (*p* = 0.0276, Wilcoxon matched-pairs test) and saline hPMCA2w/b (*p* = 0.0056, Wilcoxon matched-pairs test), but not in cocaine tdTomato or cocaine hPMCA2w/b groups. Effect sizes were 0.50, 0.41, 0.20, and 0.18 for saline tdTomato, saline hPMCA, cocaine tdTomato, and cocaine hPMCA2w/b groups, respectively (Figure 6D). The frequency of neuronal Ca^2+^ events was significantly suppressed by NPS-2143 in all four groups (*p* < 0.0001, Wilcoxon matched-pairs tests) with effect sizes of 2.02, 1.65, 2.19, 1.65 for saline tdTomato, saline hPMCA2w/b, cocaine tdTomato, and cocaine hPMCA2w/b groups, respectively (Figure 6E). These results indicate that while cocaine self-administration may alter the impact of CaSR inhibition on duration of neuronal Ca^2+^ transients, this effect is independent of hPMCA2w/b expression. Moreover, neither the cocaine self-administration nor hPMCA2w/b expression altered NPS-2143 effect on amplitude and frequency of neuronal Ca^2+^ events. We conclude that increased extracellular Ca^2+^ that may arise secondary to over-expression of hPMCA2w/b in striatal astrocytes, does not account for elevated neuronal activity in hPMCA2w/b treated animals trained to self-administer cocaine.

## Discussion

Our findings highlight the changes in neuronal Ca^2+^ signaling and behavioral responses to cocaine that result from suppression of astrocyte Ca^2+^ in the dorsal striatum. We observe two main results: increased cue-induced reinstatement of cocaine-seeking and increased amplitude of neuronal Ca^2+^ events. With regard to behavior, our findings are consistent with the reports demonstrating that following a period of abstinence, G_q_-DREADD-mediated activation of astrocytes in the nucleus accumbens core reduced drug-seeking behaviors, presumably via mechanisms that involve elevation of astrocyte Ca^2+^ (Bull et al., 2014; Scofield et al., 2015). A prior report also indicated that suppression of astrocyte Ca^2+^ by hPMCA2w/b in the dorsal striatum triggers repetitive behaviors in mice (Yu et al., 2018). Locomotor stereotypies may accompany development of psychostimulant self-administration (Chinen et al., 2006; Hadamitzky et al., 2012) supporting our observation of increased reinstatement of cocaine seeking in hPMCA2w/b animals. It is unclear if there exists a discrete value of astrocyte Ca^2+^ below which reinstatement is more likely. The relationship between astrocyte Ca^2+^ and reinstatement may not be linear and may involve processes that were not evaluated in the current study, such as extent of astrocyte gap-junction coupling. Further insight could be gained by establishing that cue-induced reinstatement scales with the hPMCA2w/b viral titer and by correlating astrocyte Ca^2+^ concentration with neuronal Ca^2+^ activity as a function of hPMCA2w/b titer. Since Ca^2+^ wave propagation between wild-type astrocytes is clearly operative in the striatum (e.g., Supplementary video 1), any numerical estimate of astrocyte Ca^2+^ concentrations sufficient to sustain “normal” vs. “cocaine-oriented” behavior would likely also have to include quantitative analysis of hPMCA2w/b abundance both within and in proximity to imaged cells.

Interestingly, our results show that the magnitude of hPMCA2w/b (relative to tdTomato) effect on active lever presses after cocaine self-administration training is larger on the last day of FR1 training (ratio = 1.44) than on the last day of FR3 (ratio = 1.29) training. While the transition to FR3 represents only a marginal elevation in the amount of effort required to obtain cocaine infusions, this difference raises the possibility of astrocyte Ca^2+^ impact on motivation for cocaine that could be further explored with a progressive ratio task. Indeed, activation of G_q_ signaling in the nucleus accumbens core astrocytes has been found to increase motivation to self-administer ethanol (Bull et al., 2014). Lever press behavior during extinction was not different between groups in our experiments, although prior research has shown that extinction of drug-seeking is a critical period during which changes in astrocyte morphology and astrocyte co-localization with synapses in the NAc and ventral pallidum may emerge (Scofield et al., 2016; Testen et al., 2018; Kruyer et al., 2022). It is not clear whether structural changes in dorsal striatum astrocytes contribute to our finding that suppression of astrocyte Ca^2+^ promotes increased neuronal activity after cocaine self-administration training. Both closer and greater distance between astrocyte and neuronal processes may result in increased neuronal activity, for example, via increased sensitivity to Ca^2+^-dependent release of astrocyte glutamate at neuron-proximal sites or via reduced glutamate uptake in the case of increased separation between astrocyte and neuronal processes. Additionally, astrocyte activity has been noted to regulate neuronal morphology (Blanco-Suarez et al., 2017), although we did not observe differences in neuronal appearance at the gross morphological level in our experiments. Notably, the original manuscript describing generation of the hPMCA2w/b virus (Yu et al., 2018), eliminated astrocyte activation as the mechanism underlying hPMCA2w/b-mediated suppression of astrocyte Ca^2+^. Detailed investigations of how suppression of astrocyte Ca^2+^ may impact fine details of astrocyte and neuronal morphology remain to be conducted.

With regard to cellular changes, reducing astrocyte Ca^2+^ signaling has been previously shown to impact MSN activity. In awake, freely behaving mice, hPMCA2w/b expression was associated with reduced amplitudes, shorter durations, but unchanged frequencies, of neuronal Ca^2+^ signals (Yu et al., 2018). We observed a non-significant decrease of neuronal Ca^2+^ amplitude in our data (saline SA tdTomato vs. saline SA hPMCA2w/b group in Figure 4). This discrepancy could be due to species or other methodological differences but could also result from exposure to the operant chamber environment, that may have effects independent from cocaine exposure (O'Donovan et al., 2021). Our data show that cocaine-induced reduction in the amplitude of neuronal Ca^2+^ events is rescued by hPMCA2w/b over-expression in cocaine-trained animals. Notably, hPMCA2w/b over-expression in animals trained to self-administer saline had no effect on neuronal activity, indicating that astrocyte Ca^2+^ interaction with neuronal signaling required cocaine self-administration experience. Cocaine self-administration training leads to numerous molecular adaptations that may impact neuronal Ca^2+^, including a wide range of gene expression changes (Freeman et al., 2010; Walker et al., 2018; Wang Y. et al., 2021), altered membrane excitability (Schramm-Sapyta et al., 2006; Wang Y. et al., 2021; He et al., 2023) and glutamatergic synapse activity (Schramm-Sapyta et al., 2006; Wang Y. et al., 2021; Catalfio et al., 2023). Plasticity of neuronal NMDA receptors, particularly those at extrasynaptic sites is of particular relevance for detection of astrocyte-released glutamate after cocaine exposure (Self et al., 2004; Yamamoto and Zahniser, 2012; Ortinski et al., 2013; Ortinski, 2014; O'Donovan et al., 2021). Activation of Ca^2+^-permeable neuronal NMDA receptors by astrocyte-derived glutamate generates characteristically slow neuronal currents that have been found to occur more frequently in the NAc neurons after cocaine self-administration training without extinction (O'Donovan et al., 2021). Our finding that hPMCA2w/b over-expression impacts the duration of neuronal Ca^2+^ transients may reflect an interaction between Ca^2+^-dependent glutamate release from astrocytes and extrasynaptic NMDA receptors in the dorsal striatum that continues through or emerges during the extinction training phase. However, hPMCA2w/b impact on duration of neuronal Ca^2+^ events may also be linked to dynamics of neuronal Ca^2+^ entry or buffering via mechanisms not related to glutamate receptor channels. Although many studies have demonstrated that Ca^2+^ signaling in astrocytes facilitates release of astrocyte glutamate, others have argued against Ca^2+^-dependent gliotransmitter release (Savtchouk and Volterra, 2018) and our data do not address whether Ca^2+^-induced glutamate release from astrocytes is necessary for regulation of neuronal activity that we observe. Although the potential role of Ca^2+^-dependent release of glutamate from astrocytes is intriguing, given the well-documented impact of glutamate on cocaine-seeking behavior (Kalivas, 2009; Knackstedt and Kalivas, 2009; Scofield et al., 2015), contribution of other astrocyte-secreted neurotransmitters, such as ATP or GABA (Losi et al., 2014) should not be overlooked. Overall, our findings of hPMCA2w/b effect on neuronal output are consistent with observations that excitatory signaling in the dorsal striatum is required for cue-induced cocaine seeking (Vanderschuren et al., 2005), cue-induced reinstatement (Gabriele and See, 2011) and context-induced reinstatement (Fuchs et al., 2006).

Given distinct effects of astrocyte Ca^2+^ on neurons from saline and cocaine self-administration groups, we sought to evaluate the consequences of hPMCA2w/b overexpression on neuronal response to acute cocaine. We found that acute cocaine broadly inhibited amplitude and frequency of neuronal Ca^2+^ transients. This parallels findings of cocaine-induced suppression of field potentials in corticostriatal slices (Chiodi et al., 2014) and reduction in frequency of Ca^2+^ transients in the NAc during fiber photometry recordings *in vivo* (Calipari et al., 2016). In our experiments, inhibition of neuronal activity by cocaine was strongest in tdTomato animals with a history of cocaine self-administration as measured by effect sizes in acute cocaine experiments (Figures 5C_ii_, E_ii_), suggesting sensitization to pharmacological effects of cocaine. It is not clear whether this neuronal sensitization is related to the widely reported locomotor sensitization after repeated cocaine intake. Recent evidence suggests that locomotor sensitization may depend on cocaine-induced baseline neuronal hypoactivity (Kwon et al., 2023; Wang et al., 2023), a phenomenon that we also observe in our Ca^2+^ imaging data (Figure 4B).

How does cocaine self-administration impact astrocyte Ca^2+^ to regulate neuronal activity? In astrocytes, acute cocaine administration has been shown to increase Ca^2+^ event frequency in the nucleus accumbens (Wang J. et al., 2021), similar to the effects of acute dopamine in cultured hippocampal astrocytes (Galloway et al., 2018). In neurons, our findings are consistent with suppression of field potentials in corticostriatal slices by acute cocaine (Chiodi et al., 2014), suppression of neuronal firing during cocaine self-administration (Coffey et al., 2015) and D1 receptor-mediated hyperpolarization of medium spiny neurons by acute dopamine (Uchimura and North, 1990). However, D2-mediated depolarization of membrane potential by dopamine has also been reported (Uchimura and North, 1990) and may involve synergistic interaction with D1-receptors that promotes action potential firing (Hopf et al., 2003). In general, acute cocaine or dopamine allow for bi-directional regulation of neuronal activity depending on the complement of ion channels available at a particular cell, but many mechanistic questions, including the effects of possible interactions between these channels, remain unresolved even after decades of research (Nicola et al., 2000).

An important consideration in probing the cellular mechanisms that underlie interaction between astrocyte Ca^2+^ and neuronal activity in the hPMCA2w/b model is the impact of potentially elevated extracellular Ca^2+^ as a result of extrusion by the over-expressed hPMCA2w/b. We evaluated this by measuring the impact of CaSR inhibition on neuronal Ca^2+^ signals. CaSR transcripts have been identified in both the gray and white matter within subpopulations of neurons and oligodendrocytes, but not with astrocytes or microglial markers (Mudò et al., 2009; Ruat and Traiffort, 2013). CaSRs are G-protein-coupled receptors, sensitive to extracellular Ca^2+^ in the high-nanomolar to low millimolar range that have been implicated in various physiological functions (Mudò et al., 2009). Binding of extracellular Ca^2+^ is thought to activate the G_q_/11 pathway, leading to Ca^2+^ release from intracellular stores (Lu et al., 2010; Thakker, 2012; Abid et al., 2021). If hPMCA2w/b expression facilitated neuronal Ca^2+^ transients by raising extracellular Ca^2+^ levels, we would expect a stronger effect of NPS-2143 in hPMCA2w/b relative to the tdTomato groups. Instead, we found that although inhibition of endogenous CaSR did decrease amplitude and frequency of neuronal Ca^2+^ events, it did not result in a unique pattern of inhibition in experimental groups that over-expressed hPMCA2w/b. These observations suggest that the relationship between suppressed astrocyte Ca^2+^ and increased neuronal activity after cocaine self-administration is not driven by increased extracellular Ca^2+^ levels. Instead, our evidence supports the interpretation that astrocyte Ca^2+^ impacts cell-intrinsic mechanisms, such as release of neuroactive molecules from astrocytes, astrocyte regulation of metabolic pathways (e.g. glutamate-glutamine cycle), or morphological changes that alter astrocyte-neuron interactions (Martín et al., 2015; Testen et al., 2018; Piroli et al., 2019). Clearly, changes in CaSR expression may impact this interpretation. For example, CaSR gene expression was found to decrease after exposure of cultured human fetal cells to cocaine (Lee et al., 2009). A similar effect at the protein level in the striatum may have reduced the magnitude of NPS-2143-induced inhibition of neuronal Ca^2+^ in our cocaine self-administering animals.

Our astrocyte Ca^2+^ suppression strategy involved both dorsolateral and dorsomedial striatum subregions based on literature that such targeting influenced behavioral stereotypies (Yu et al., 2018) and habit formation (Kang et al., 2020) that could be relevant to cocaine seeking. A long-standing theory postulates a unique role for dorsolateral striatum activity in encoding habitual action, whereas dorsomedial striatum circuits maintain goal-directed action (Balleine et al., 2009). The theory is complicated by findings that goal-directed action encoding is uniquely localized to the posterior, but not the anterior, aspect of dorsomedial striatum (Yin and Knowlton, 2004) and directly challenged by observations that habitual action cannot be attributed to unique differences between dorsolateral and dorsomedial striatum neuronal activity (Vandaele et al., 2019). Nevertheless, circuit-specialization of astrocyte Ca^2+^ signals has been theorized in the dorsal striatum (Martín et al., 2015) and differences in impact of astrocyte Ca^2+^ on the output of dorsomedial and dorsolateral striatum neurons could lend support to this theory.

We acknowledge certain limitations within our work. For example, while our experiments included both male and female rats to avoid sex-biased results, they were not powered to specifically detect sex differences. With this caveat in mind, we did detect significant differences between males and females as follows: cocaine tdTomato females pressed less than cocaine tdTomato males on the last day of FR1 and FR3, saline tdTomato females had smaller Ca^2+^ transient amplitudes than saline tdTomato males, and cocaine hPMCA2w/b females had smaller frequency of Ca^2+^ transients than cocaine hPMCA2w/b males. Further experimentation is necessary to confirm male/female differences with sufficient statistical power. Another caveat is that the GCaMP6f Ca^2+^ indicator strategy that we employ does not distinguish between D1-like and D2-like MSNs, despite the unique roles of these cell types in cocaine-induced plasticity (Luo et al., 2011; Smith et al., 2013; Ortinski et al., 2015; Calipari et al., 2016; Pascoli et al., 2023). Finally, our study focused on cue-induced reinstatement of cocaine-seeking. Additional behavioral paradigms, measuring the impact of astrocyte Ca^2+^ on affective or cognitive effects would certainly provide a more nuanced understanding of astrocyte contribution to behavioral outcomes of drug use.

## Data Availability

The raw data supporting the conclusions of this article will be made available by the authors, without undue reservation.

## Supplementary material

Supplementary material 1 Raw unprocessed videos demonstrating astrocyte GCaMP6f fluorescence in slices from tdTomato (video 1) and hPMCA2w/b animals (video 2). Frame numbers are indicated in the upper left. Original videos were acquired at 25 Hz but are presented at 125 Hz for illustration. Arrows in the hPMCA2w/b video 2 highlight two astrocytes with brief periods of activity (e.g., left arrow: frames 950–970; right arrow: frames 1042–1087). Raw unprocessed videos demonstrating neuronal GCaMP6f fluorescence in slices from tdTomato and hPMCA2w/b animals are in videos 3-7. aCSF_M4 video 3 corresponds to the field of view in Figure 4B. aCSF_M5 video 4 corresponds to the field of view in Figure 5B; cocaine_M5 video 5 is the same field of view after bath application of cocaine, corresponding to Figure 5C. aCSF_M4 video 6 is the field of view in Figure 6B; NPS_F9 video 7 is the same field of view after bath application of NPS-2143, corresponding to Figure 6C. Note that the onset of neuronal fluorescence is near instantaneous rather than spreading through the soma and syncytium territories as Ca^2+^ “waves” characteristic of astrocyte signals. In cases where neuronal processes are captured in the imaging plane, fluorescence in neuronal processes always accompanies fluorescence in the soma they are connected to. This is not the case for astrocyte Ca^2+^ where soma and process fluorescence can be independent of each other.

Supplementary Figure 1 Minimal impact of astrocyte Ca^2+^ on inactive lever press responses. **(A)** Inactive presses across all days of self-administration, and extinction training. **(B)** Histogram of inactive lever presses during the final day of FR1 training. **(C)** Histogram of inactive lever presses during the final day of FR3 training. **(D)** Histogram of inactive lever presses during the last day of extinction. (**E)** Histogram of inactive lever presses during cue-induced reinstatement testing. *N* = 6–7 animals in each group. ^*^*p* < 0.05, main effect of cocaine, two-way ANOVA. All data are plotted as mean ± SEM.

Supplementary Figure 2 Lack of correlation between active lever press numbers during FR3 and reinstatement testing. **(A)** Active lever presses (excluding time-out presses) for the last day of FR3 and corresponding total reinstatement presses for each animal. **(B)** Active lever presses (including time-out presses) for the last day of FR3 and corresponding total reinstatement presses. **(C)** Same as A), except split between tdTomato and hPMCA2w/b groups. **(D)** Same as **(B)**, except split between tdTomato and hPMCA2w/b groups. *N* = 6–7 animals in each group. Pearson's correlation coefficients (r) along with the corresponding *p*-values are indicated.
