# Supplementary figures and images for: Astrocyte Ca2+ in the dorsal striatum suppresses neuronal activity to oppose cue-induced reinstatement of cocaine seeking
Source: Front Cell Neurosci. 2024 Aug 29;18:1347491. doi: 10.3389/fncel.2024.1347491 (PMC11393831; doi:10.3389/fncel.2024.1347491)

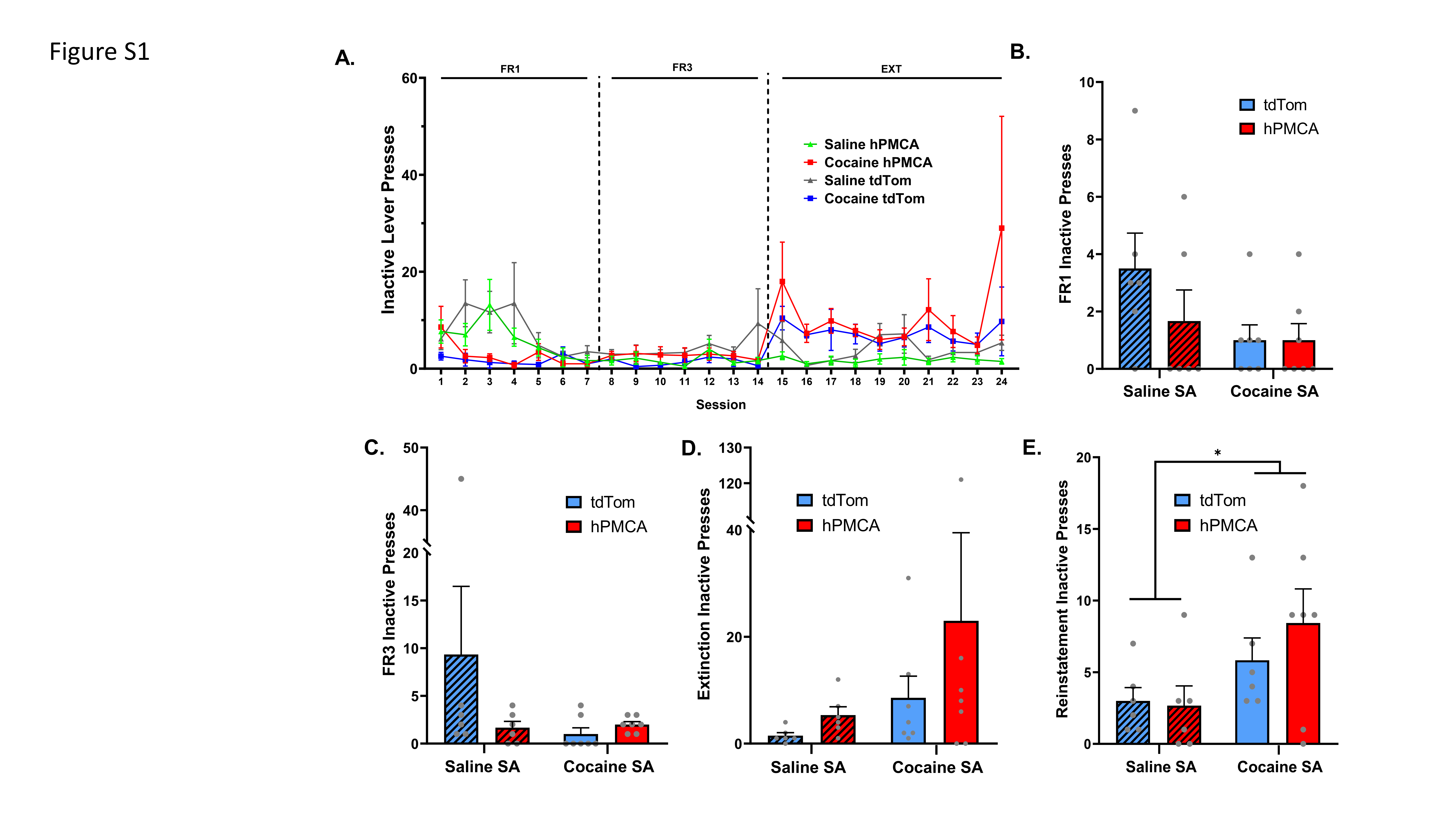

Supplement: Supplementary Figure 1 — Minimal impact of astrocyte Ca2+ on inactive lever press responses. (A) Inactive presses across all days of self-administration, and extinction training. (B) Histogram of inactive lever presses during the final day of FR1 training. (C) Histogram of inactive lever presses during the final day of FR3 training. (D) Histogram of inactive lever presses during the last day of extinction. (E) Histogram of inactive lever presses during cue-induced reinstatement testing. N = 6–7 animals in each group. *p < 0.05, main effect of cocaine, two-way ANOVA. All data are plotted as mean ± SEM. [file Image_1.tif]

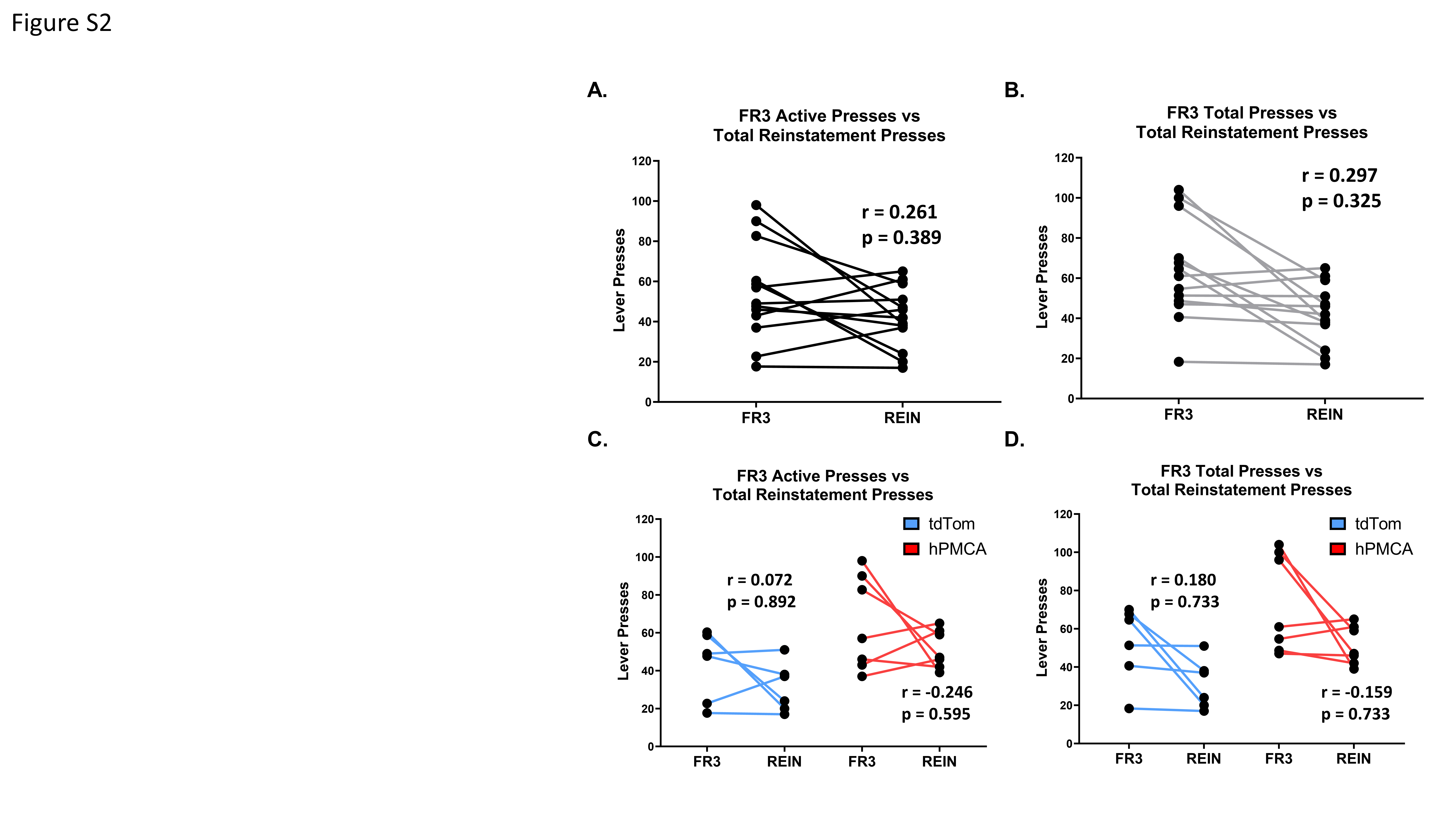

Supplement: Supplementary Figure 2 — Lack of correlation between active lever press numbers during FR3 and reinstatement testing. (A) Active lever presses (excluding time-out presses) for the last day of FR3 and corresponding total reinstatement presses for each animal. (B) Active lever presses (including time-out presses) for the last day of FR3 and corresponding total reinstatement presses. (C) Same as A), except split between tdTomato and hPMCA2w/b groups. (D) Same as (B), except split between tdTomato and hPMCA2w/b groups. N = 6–7 animals in each group. Pearson's correlation coefficients (r) along with the corresponding p-values are indicated. [file Image_2.tif]
